# Supplementary material for: Correlation and Difference between Core Micro-Organisms and Volatile Compounds of Suan Rou from Six Regions of China
Source: Foods. 2022 Sep 5;11(17):2708. doi: 10.3390/foods11172708 (PMC9455853; doi:10.3390/foods11172708)
Supplement: Supplementary file 1 [file foods-11-02708-s001.zip › foods-1893993-supplementary.pdf]

Supplementary Materials

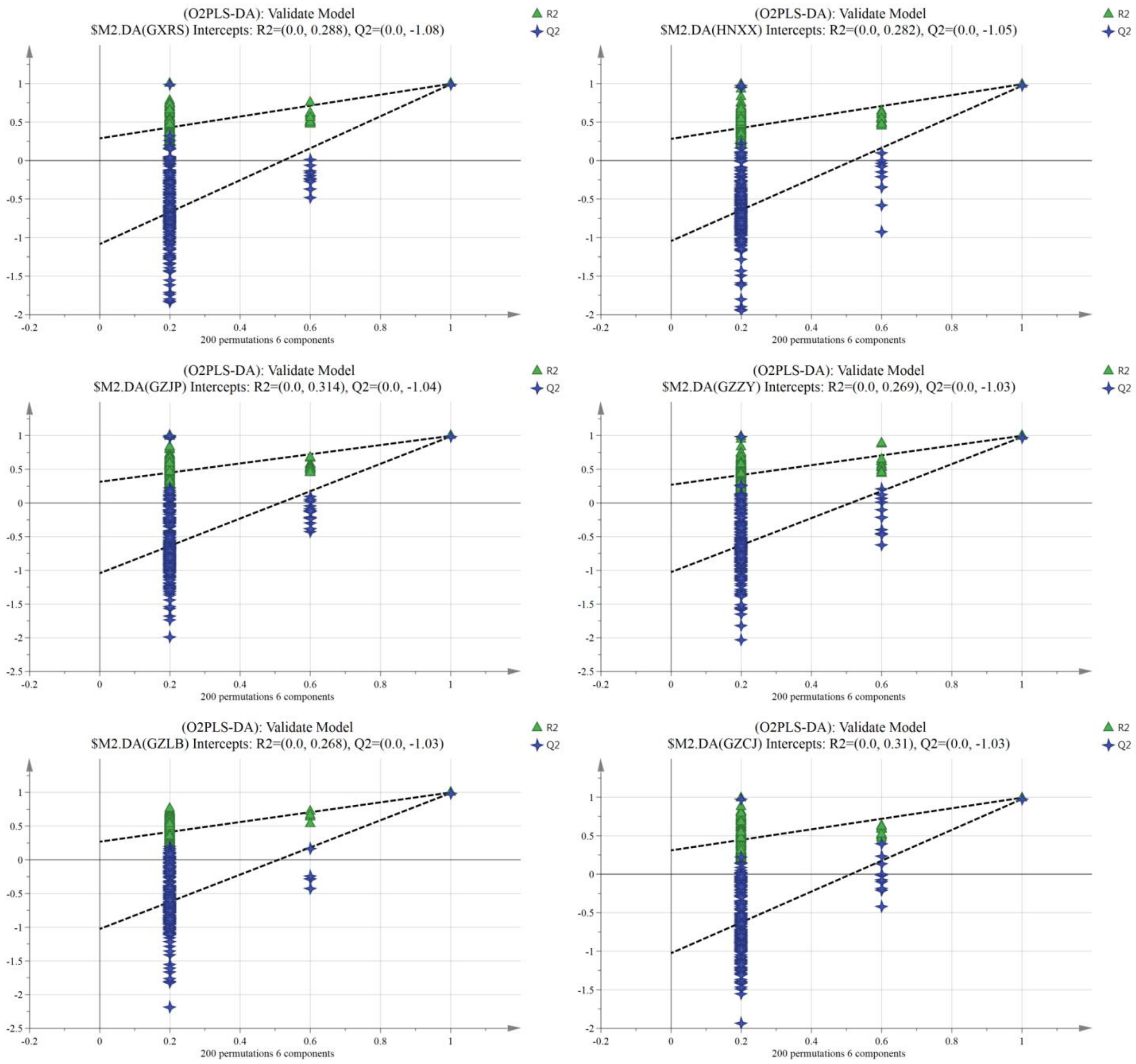

Figure S1. The Permutations Plot for O2PLS-DA. The triangle represents R2, the four-pointed star represents Q2, and the dotted line represents their respective regression lines.
